# An Extensive Lymphatic Malformation of the Parotid and Parotideo-Masseteric Region in an Adolescent: Nerve-Preserving Surgical Management and Postoperative Imaging Follow-Up: A Case Report

**DOI:** 10.3390/children13081070

**Published:** 2026-08-12

**Authors:** Michał Gontarz, Krzysztof Gąsiorowski, Krystyna Gałązka, Tomasz Marecik, Jakub Bargiel, Paweł Szczurowski, Katarzyna Rusek, Grażyna Wyszyńska-Pawelec

**Affiliations:** 1 Department of Cranio-Maxillofacial Surgery, Jagiellonian University Medical College, 30-688 Cracow, Poland; krzysztof.gasiorowski@uj.edu.pl (K.G.); tomasz.marecik@uj.edu.pl (T.M.); jakub.bargiel@uj.edu.pl (J.B.); pawel.szczurowski@uj.edu.pl (P.S.); krusek@su.krakow.pl (K.R.); grazyna.wyszynska-pawelec@uj.edu.pl (G.W.-P.); 2 Department of Pathomorphology, Jagiellonian University Medical College, 30-688 Cracow, Poland; krystyna.galazka@uj.edu.pl

**Keywords:** lymphatic malformation, lymphangioma, parotid gland, parotidectomy, facial nerve, adolescent, vascular anomaly, cystic hygroma, surgical management, magnetic resonance imaging follow-up

## Abstract

**Highlights:**

**What are the main findings?**

- Extensive lymphatic malformation of the left parotid–masseteric region in a 16-year-old, reaching the deep parotid lobe and parapharyngeal space, was treated by nerve-sparing partial parotidectomy (ESGS I, II, IV) with full facial nerve preservation.
- Despite a postoperative sialocele requiring reintervention, MRI at 15 months showed only residual disease without progression.

**What are the implications of the main findings?**

- Function-preserving parotidectomy with neuromonitoring effectively controls extensive parotid lymphatic malformations while sparing the facial nerve.
- Given infiltrative growth near critical structures, long-term MRI surveillance is preferable to aggressive re-resection.

**Abstract:**

**Background:** Lymphatic malformations of the parotid gland and of the adjacent parotideo-masseteric region are rare and technically demanding, because the malformation infiltrates the glandular parenchyma and grows around the branches of the facial nerve rather than displacing them, and published experience consists almost entirely of isolated case reports. **Case Presentation:** A 16-year-old boy was treated for an extensive lymphatic malformation of the left parotid and parotideo-masseteric region, present since childhood and progressive over three years, with facial asymmetry, no pain and normal facial nerve function. Magnetic resonance imaging showed a mixed lesion of 47 × 20 × 55 mm involving the superficial and deep lobe with parapharyngeal extension. Facial nerve-preserving partial parotidectomy of European Salivary Gland Society levels I, II and IV was performed under continuous nerve monitoring. Histology confirmed a cavernous lymphangioma (CD31 and D2-40 positive). A postoperative sialocele was managed after a single revision. **Conclusions:** Level-classified nerve-preserving parotidectomy with monitoring was associated with stable residual disease and preserved facial nerve function during 19 months of follow-up. The radial resection margin was focally involved and a thin residual lesion persisted on imaging. Stability at follow-up does not establish the absence of future progression, and longer follow-up is required.

## 1. Introduction

Lymphatic malformations are slow-flow vascular anomalies that are classified as simple malformations of lymphatic origin within the classification of the International Society for the Study of Vascular Anomalies [1]. They arise from somatic activating variants of the phosphatidylinositol 3-kinase pathway, most often in PIK3CA, in lymphatic endothelial precursors [2]. The phenotypic spectrum, diagnostic criteria and testing eligibility of PIK3CA-related overgrowth are increasingly well defined, which bears directly on the use of pathway-directed therapy [3,4]. Involvement of the parotid gland is distinctly uncommon. Vascular anomalies account for a small fraction of pediatric parotid masses, and the published experience with lymphatic malformation at this site consists almost entirely of single cases [5,6,7,8,9].

Two anatomical features separate the parotideo-masseteric region from the lateral neck, where percutaneous sclerotherapy has become the first-line intervention. The facial nerve is not displaced by the lesion but runs between and around its components, so that dissection proceeds along the epineurium through abnormal and friable tissue, a relationship documented in both operative reports and high-resolution imaging [6,9,10]. The parotid capsule and fascia are frequently infiltrated, so that the plane which permits extracapsular dissection of a benign parotid tumor is absent and removal requires sacrifice of glandular parenchyma [7]. A lesion that also crosses the stylomandibular tunnel adds a parapharyngeal component in which the distribution of a sclerosant is unpredictable.

Reports addressing this site rarely combine standardized operative nomenclature, documented facial nerve outcome and postoperative imaging, and follow-up seldom exceeds six months. The specific knowledge gap addressed here is the management of a mixed parotid lymphatic malformation involving both the superficial and the deep lobe with parapharyngeal extension, reported with a standardized description of the extent of parotid resection, an objective facial nerve outcome and postoperative imaging follow-up. The present report describes an extensive lymphatic malformation of the left parotid and parotideo-masseteric region in a 16-year-old patient treated by facial nerve-preserving parotidectomy.

## 2. Case Presentation

A 16-year-old boy was admitted in December 2024 for surgical treatment of a lymphatic malformation of the left parotid gland and of the parotideo-masseteric region. The lesion had been noted in early childhood and followed without intervention. The malformation had never been treated surgically or by sclerotherapy. Body mass index was 16.67 kg/m^2^, and there was no reported family history of vascular anomalies or overgrowth syndromes. Over the preceding three years it enlarged progressively and produced a visible facial asymmetry. The patient reported no pain and scored 0 on a 0 to 10 numerical rating scale. He described no episode of sudden enlargement, no intralesional bleeding, no infection and no airway symptoms, and no previous treatment, medication, allergy or illness.

Examination showed a soft, non-tender, poorly demarcated swelling of the left parotid and cheek region without skin discoloration. Facial nerve function was normal in all branches, corresponding to House-Brackmann grade I. There was no trismus, no oropharyngeal bulging and no cervical lymphadenopathy. The lesion was unilateral and suprahyoid, corresponding to stage II of the staging system of de Serres [11]. Progressive enlargement with increasing facial asymmetry during adolescence was considered an indication for active treatment rather than continued observation. Serial objective measurements from earlier years were not available. Progression was documented clinically as a visible increase in facial asymmetry over three years.

Before surgery the case was discussed with an interventional radiologist. Percutaneous sclerotherapy was not formally considered a suitable option, because the infiltrative architecture of the lesion, with malformed channels intermingled with salivary parenchyma and surrounding the branches of the facial nerve, precluded uniform distribution of a sclerosant and carried a risk of injury to the facial nerve. Surgical resection was therefore selected as the primary treatment (Table 1).

**Figure 1:**
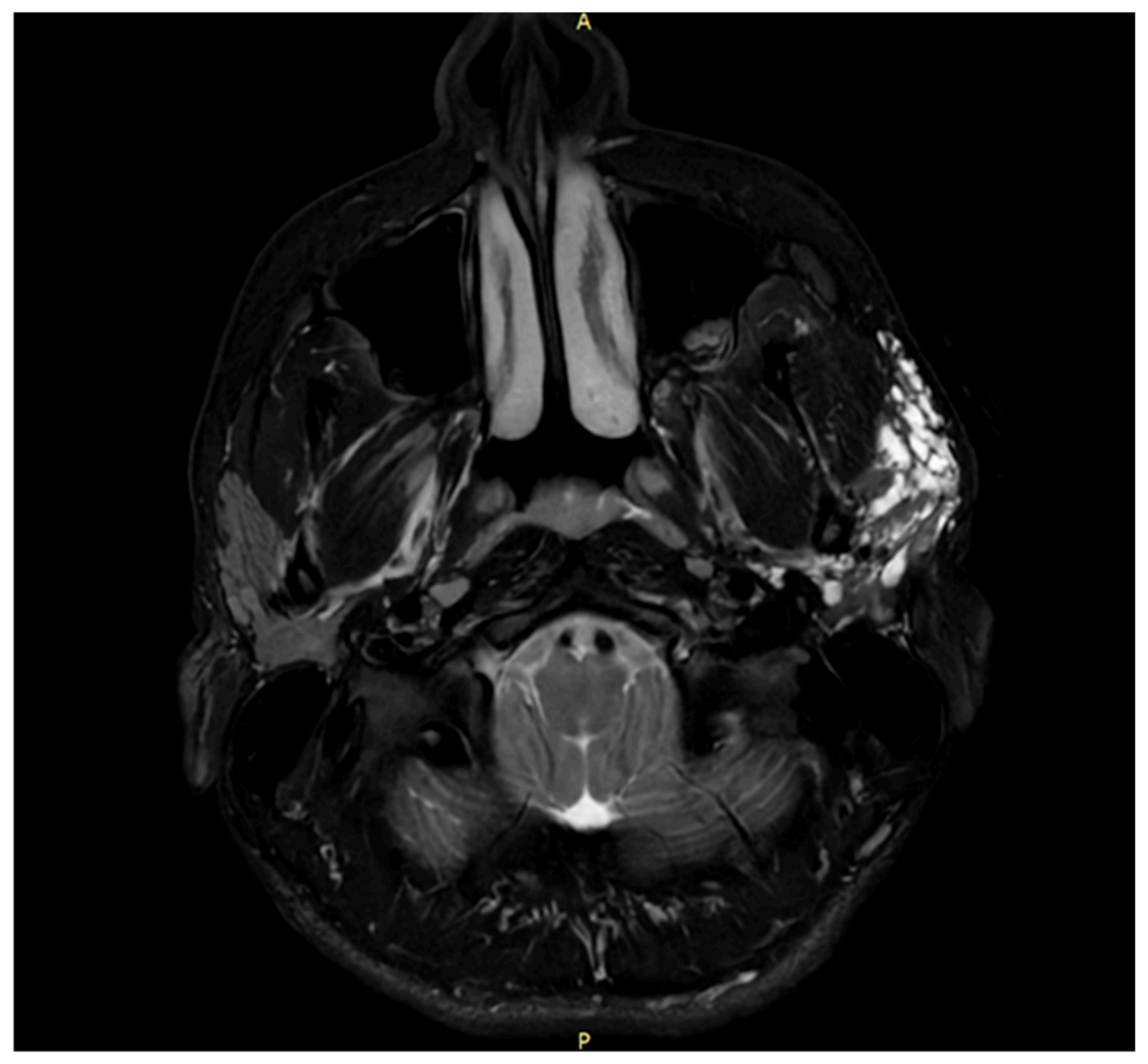
Axial T2-weighted fat-saturated magnetic resonance image before treatment. An extensive lymphatic malformation occupies the left parotideo-masseteric region and the parotid gland, showing irregular walls and high signal content, and extending medially towards the parapharyngeal space.

Magnetic resonance imaging demonstrated a lesion of fluid signal intensity anterior to the upper part of the left parotid gland, within the subcutaneous tissue of the cheek, abutting the masseter muscle and involving the superficial lobe of the gland. The lesion measured 47 × 20 × 55 mm, corresponding to an estimated volume of approximately 27 mL (ellipsoid approximation, 0.52 × length × width × height). The walls were irregular and the content was hyperintense on T2-weighted sequences (Figure 1). The malformation penetrated medially towards the parapharyngeal space through the stylomandibular tunnel and reached the deep lobe. The contralateral parotid gland and both submandibular glands showed normal signal. The morphology corresponded to a mixed lesion with a dominant macrocystic component and infiltrative extensions of smaller caliber. According to accepted vascular-anomaly imaging criteria, the lesion was therefore classified as a mixed, predominantly macrocystic lymphatic malformation. Fine-needle aspiration cytology was not performed, because magnetic resonance imaging was considered diagnostic, cytology of lymphatic malformation is frequently inconclusive, and needle sampling carries a risk of intralesional bleeding and transient facial weakness. Definitive histology was instead obtained at resection.

**Figure 2:**
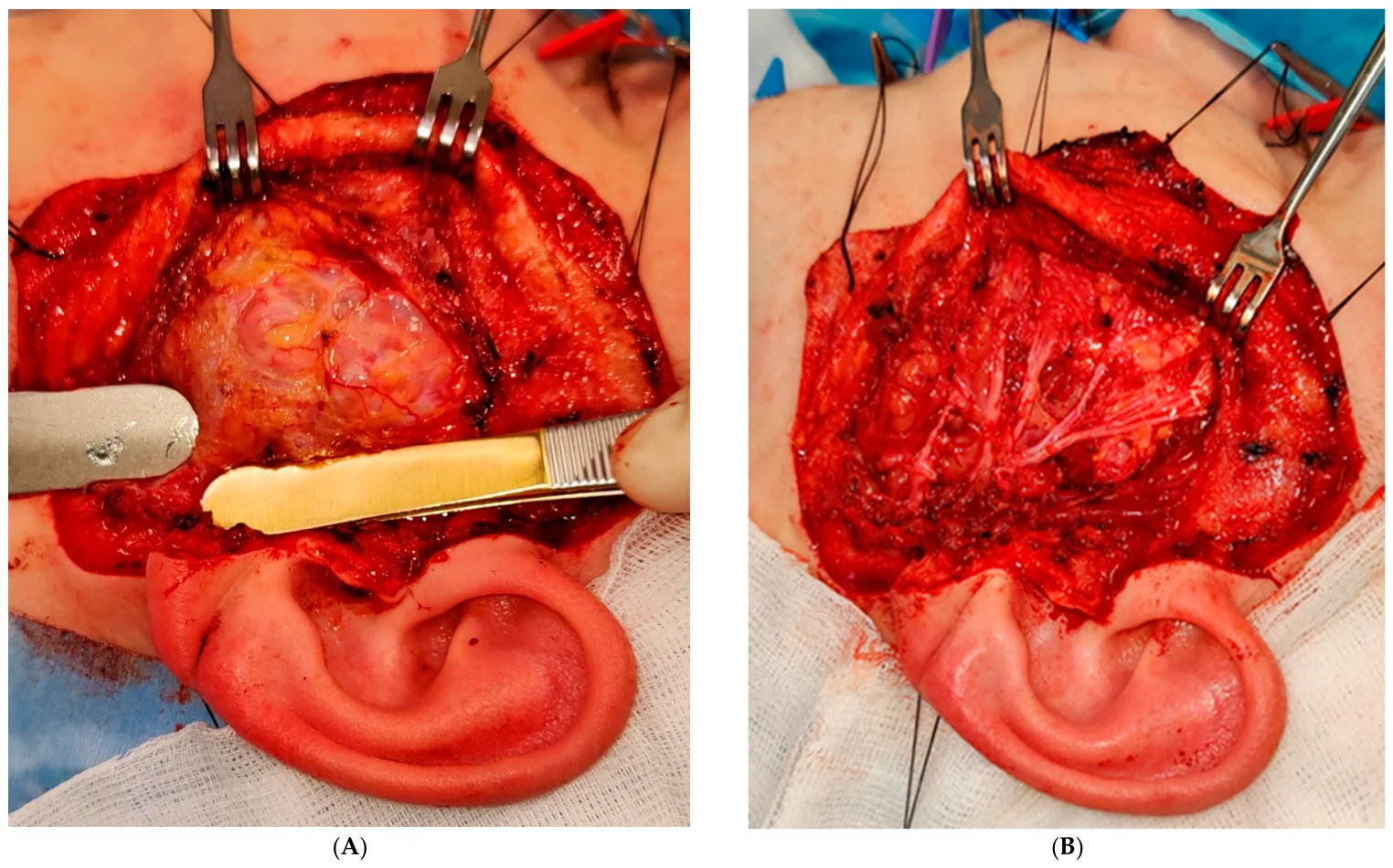
(**A**) Intraoperative view of the lymphatic malformation infiltrating the parotid fascia. (**B**) Intraoperative view after removal of the lymphatic malformation, showing preservation of the facial nerve branches.

Surgery was performed under general anesthesia without long-acting muscle relaxation, which was avoided so that continuous facial nerve monitoring remained reliable throughout the dissection. A preauricular incision with a cervical extension was used and a skin flap was raised to expose the parotid fascia, the central portion of which was infiltrated by the malformation. Parenchyma of both the superficial and the deep lobe was altered by malformed lymphatic tissue. Parotidectomy comprising levels I, II and IV of the European Salivary Gland Society classification (ESGS) was performed with removal of the malformation, using nerve stimulation throughout the dissection [12]. All branches of the facial nerve were identified within the substance of the lesion, dissected free and preserved. Stimulation at the end of the resection confirmed normal responses in every branch (Figure 2). Continuous facial nerve monitoring was performed with an Inomed C2 Nerve Monitor. A structured monitoring protocol, including stimulation parameters and baseline and final compound muscle action potential amplitudes, was not recorded. The deep-inferior compartment of the gland was preserved because it was macroscopically free of malformation and its retention did not compromise dissection of the nerve. The defect of the parotid fascia was covered with a superficial musculoaponeurotic system flap, redundant preauricular skin was excised, a suction drain was placed and the wound was closed in layers under a compressive dressing. No intraoperative complications occurred. The operative time was 195 min, with an estimated blood loss of around 70 mL.

**Figure 3:**
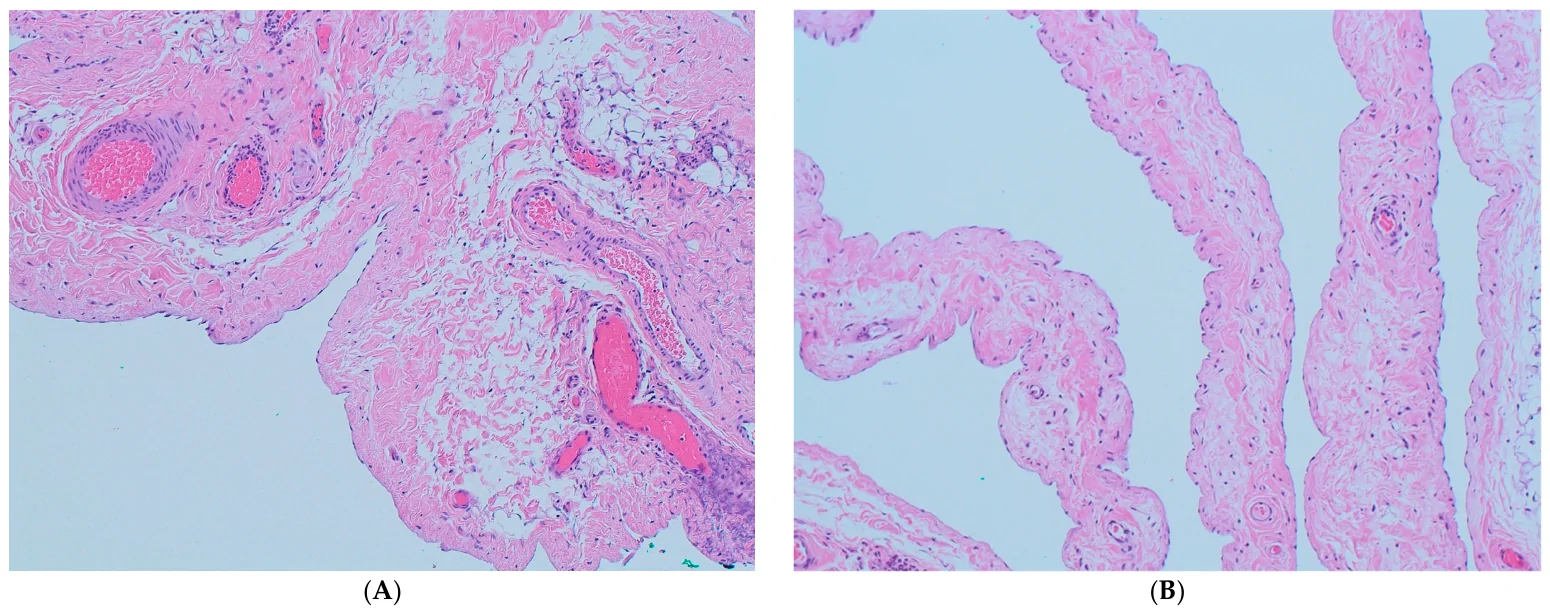

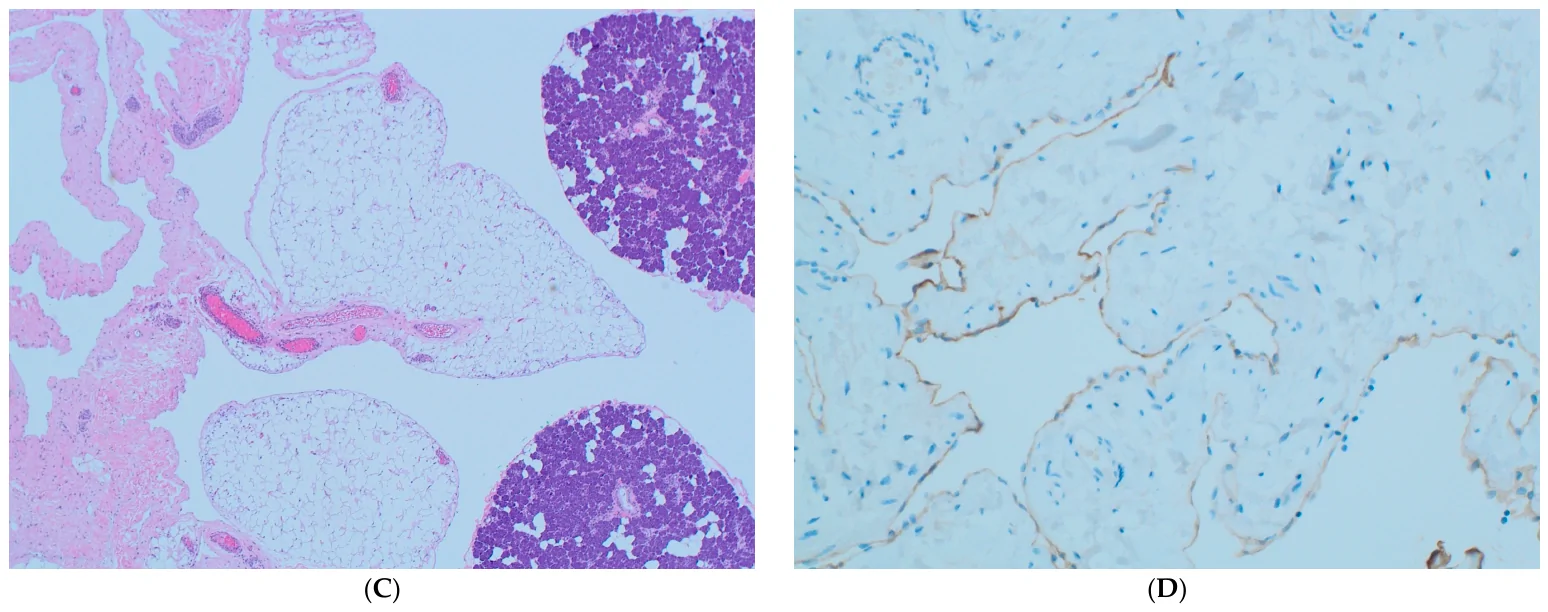
Histopathology of the resected specimen. (**A**) Peripheral part of the lesion (left) composed of wide spaces separated by thick fibrous walls, adjacent to the serous salivary parenchyma of the parotid gland (right). Haematoxylin and eosin, original magnification ×200. (**B**,**C**) Irregular empty vascular spaces of the lymphangioma with thick fibrous walls lined by flattened endothelial cells. Haematoxylin and eosin. (**D**) Expression of D2-40 (podoplanin) in the endothelial lining of the irregular lymphatic channels. Immunohistochemistry, D2-40 (DAKO-Ventana kit, Agilent Life Technologies, Dako, Glostrup, Denmark), original magnification ×400.

The specimen contained serous salivary gland tissue and adjacent fibrofatty tissue occupied by a cavernous lymphangioma with endothelial expression of CD31 and D2-40 (podoplanin), reaching the radial resection margin focally. Three lymph nodes showed no significant pathological changes. Sections demonstrated the peripheral part of the lesion composed of wide spaces separated by thick fibrous walls in the immediate vicinity of the serous salivary parenchyma, irregular empty vascular spaces lined by flattened endothelial cells, and podoplanin expression confined to that lining (Figure 3A–D).

Facial nerve function was preserved throughout, with House-Brackmann grade I in all branches. Salivary retention developed in the operative bed and formed a pseudocyst that persisted despite conservative measures. Three months after the primary resection, the pseudocyst was excised together with a fragment of the deep lobe of the left parotid gland. That specimen contained normal serous salivary parenchyma and a small focus of cavernous lymphangioma in the adjacent fibrofatty tissue, again reaching the radial margin focally. Facial nerve function remained normal and no further salivary collection occurred.

**Figure 4:**
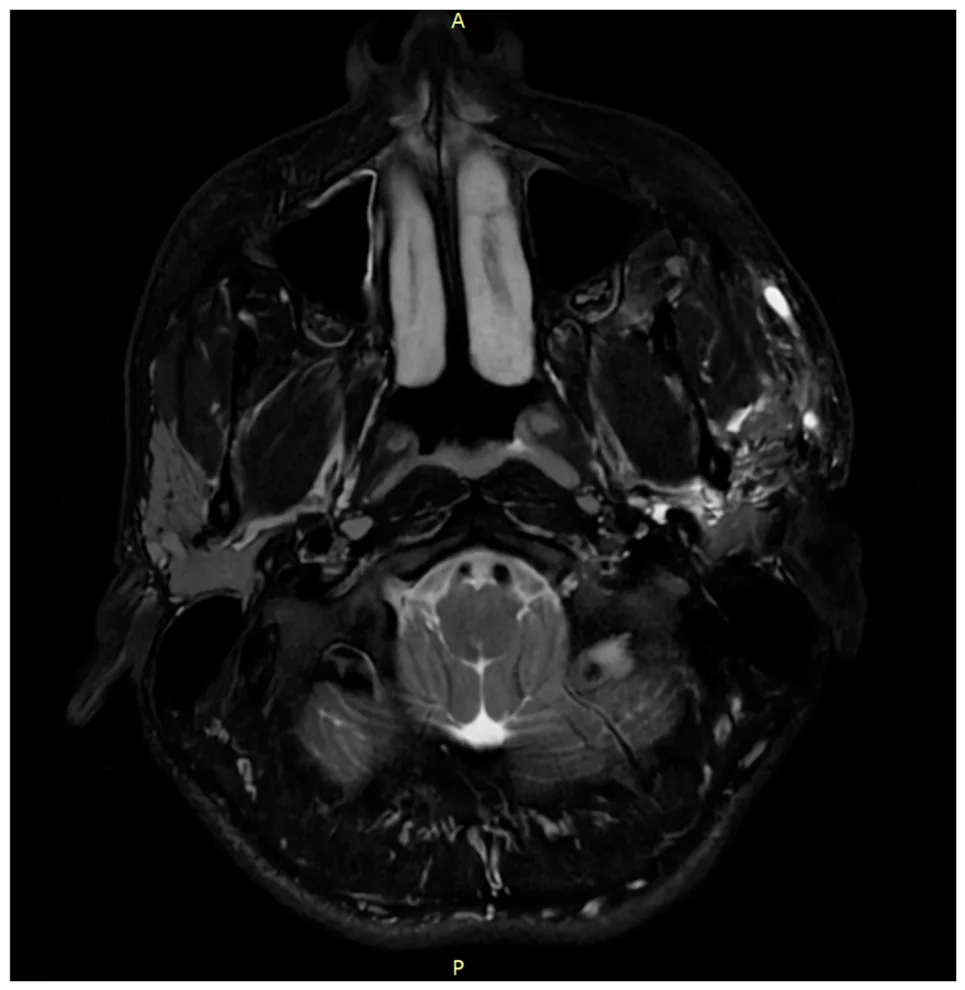
Axial T2-weighted fat-saturated magnetic resonance image 15 months after treatment. There are no features of local recurrence. Residual bands of lymphatic malformation tissue are visible in the region of the left parotid gland.

Control magnetic resonance imaging 15 months after the resection showed narrow bands of increased T2 signal along the upper contour of the gland, penetrating medially through the stylomandibular tunnel towards the parapharyngeal space, corresponding to residual tissue of the operated lymphangioma (Figure 4). There were no features of local recurrence, no expansion relative to the immediate postoperative anatomy and no new lesions.

**Figure 5:**
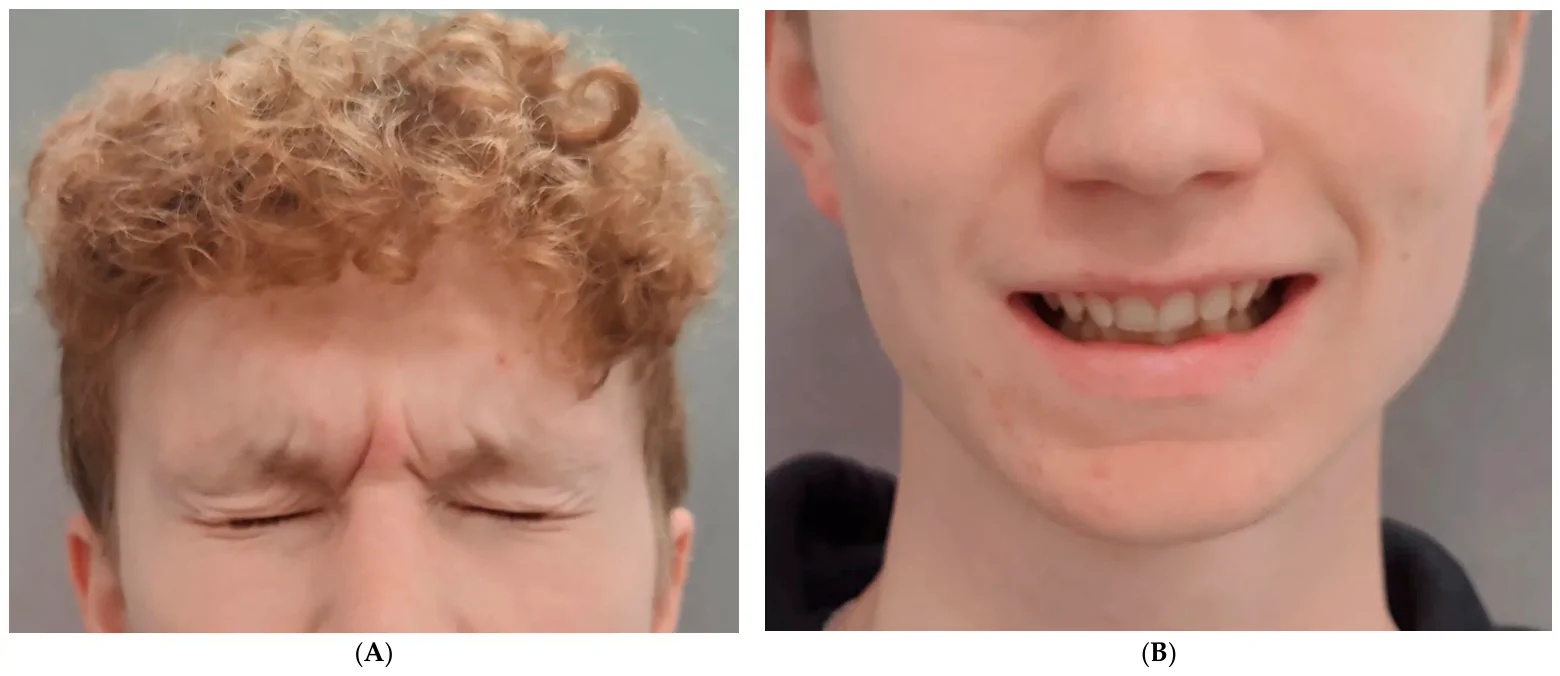

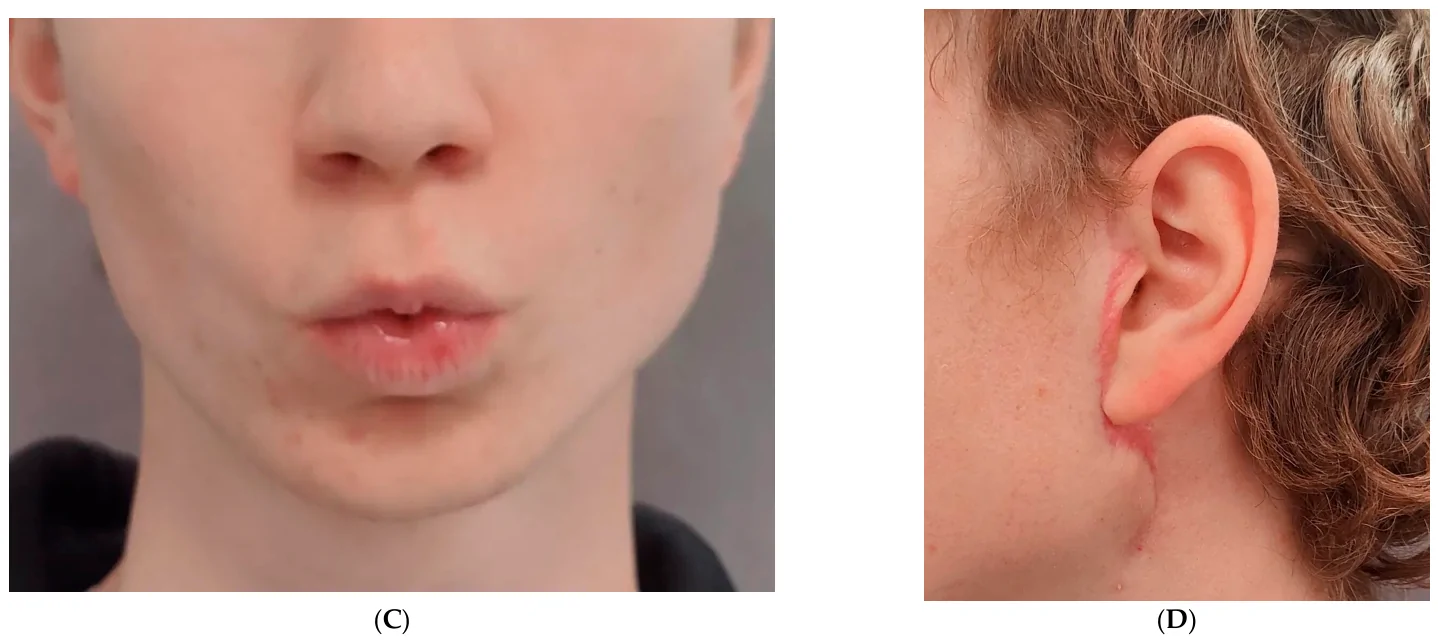
Clinical photographs at 19-month follow-up. (**A**) Symmetric elevation of the eyebrows with complete eye closure. (**B**) Symmetric smile with preserved movement of the oral commissure. (**C**) Symmetric lip pursing. Panels (**A**–**C**) document normal function of all branches of the left facial nerve, corresponding to House-Brackmann grade I. (**D**) Left preauricular region showing a hypertrophic scar along the incision.

Total follow-up was 19 months. At the most recent assessment the facial contour was symmetrical on clinical assessment. The facial nerve function was normal in all branches (House-Brackmann grade I) and was documented photographically (Figure 5A–C); no gustatory sweating was observed and the patient reported no functional complaint. The preauricular scar had become hypertrophic (Figure 5D).

## 3. Discussion

The lesion described here is termed a cavernous lymphangioma by the pathologist and a mixed lymphatic malformation by the clinician, and both descriptions refer to the same entity. This distinction is clinically important because treatment selection in the parotid region depends on cyst architecture rather than on the histological label. A malformation grows in proportion to the patient, does not involute, and expands disproportionately after intralesional bleeding, infection or hormonal change. The course in this patient, stable through childhood and progressive during adolescence, is characteristic and should be interpreted as natural behavior rather than as evidence of malignant transformation.

The clinical presentation of lymphatic malformation in the parotideo-masseteric region falls into two patterns with different surgical implications. Several published patients presented acutely, with sudden painful enlargement after minor trauma or intralesional hemorrhage, and some developed facial palsy either spontaneously or after diagnostic aspiration [6,8,9,13]. Weakness in these circumstances usually results from compression or hemorrhagic distension and resolves once the lesion decompresses [6]. The present patient followed the alternative and more indolent pattern, with slow progression, absence of pain and preserved facial nerve function. This distinction is practical rather than academic, because a lesion that has never bled or become infected is generally less adherent to the nerve and offers better conditions for elective dissection than one operated after repeated inflammatory episodes.

Magnetic resonance imaging determined the operative plan and remains the reference investigation at this site, since it defines the cystic architecture, delineates the relationship to the deep lobe and to the parapharyngeal space, and demonstrates fluid–fluid levels after intralesional hemorrhage [10]. Fine-needle aspiration cytology is frequently inconclusive, yielding lymphocytes, proteinaceous fluid and occasional salivary epithelial cells, and it carries a small risk of provoking intralesional bleeding and transient facial weakness [6,14]. The differential diagnosis of a cystic parotid mass in an adolescent includes both non-neoplastic cystic lesions and epithelial neoplasms that form cystic spaces. The cystic lesions include venous malformation, combined venolymphatic malformation, first branchial cleft anomaly, benign lymphoepithelial cyst, salivary duct cyst and plexiform neurofibroma, together with infantile hemangioma, which is uncommon at this age but remains an important pediatric parotid lesion [15,16]. The relevant neoplasms are Warthin tumor, cystadenoma, cystic pleomorphic adenoma and low-grade mucoepidermoid carcinoma. Although exceptional, malignancy can arise within a cystic parotid lesion, as in carcinoma developing from a salivary duct cyst, so that histological confirmation is mandatory rather than optional [17,18]. Residual uncertainty is resolved by immunohistochemistry, since co-expression of CD31 and of D2-40, a marker of lymphatic endothelium, confirms the lymphatic nature of the lining [19]. Additional lymphatic endothelial markers such as PROX1 and ERG were not required for diagnosis in the presence of unequivocal CD31 and D2-40 positivity. GLUT1, a marker of infantile hemangioma, was not assessed, because that diagnosis was not under consideration given the patient’s age, the imaging appearance and the unequivocal lymphatic immunophenotype.

Sclerotherapy has a defined but limited role in this region. Response depends on cyst architecture, with complete or substantial response in 94% of macrocystic lesions, 63% of mixed lesions and no response in microcystic lesions in the multicenter OK-432 trial, and low permanent morbidity across agents in a meta-analysis of 726 patients [20,21]. Facial nerve conduction after intralesional OK-432 in children with facial lymphatic malformations was normal on long-term assessment, which indicates that the nerve is not inevitably damaged by sclerosant in its vicinity [22]. Three considerations nevertheless argued against sclerotherapy as sole treatment here. The histological architecture, with wide spaces separated by thick fibrous walls and infiltrative extensions intermingled with salivary parenchyma, corresponds to a lesion in which sclerosant cannot be distributed uniformly. Injection into tissue that surrounds the branches of the facial nerve risks perineural inflammation and fibrosis, and the reassuring conduction data concern lesions of the cheek rather than intraglandular disease [22]. Sclerosant-induced fibrosis within the gland also complicates any subsequent dissection of the nerve, so that a failed course of injections may convert a difficult operation into a hazardous one. Sclerotherapy retains its place for a purely macrocystic lesion confined to the superficial lobe, for a patient in whom operation is contraindicated, and as adjuvant treatment of a residual macrocyst, including injection through the drain placed at resection. Comparative data support this division of roles, with excision and sclerotherapy achieving similar complete response rates in macrocystic disease and excision retaining an advantage in mixed lesions, and with surgical complication rates of 25 per cent against 17 per cent for sclerotherapy in a 20-year institutional series [23,24]. These comparative data are drawn from head and neck lymphatic malformation cohorts rather than from series confined to the parotid gland, so their extrapolation to intraglandular disease should be cautious. No series confined to intraparotid lymphatic malformation of comparable size exists, and the response and complication rates cited above should therefore be regarded as indicative rather than directly applicable to this anatomical location. In the present patient, after consultation with interventional radiology, these considerations led to primary surgery rather than sclerotherapy.

Where surgery is chosen, the extent of resection should be reported in standardized terms. Accounts of parotid surgery for lymphatic malformation almost invariably use the expressions superficial parotidectomy or total conservative parotidectomy, which conceal wide variation in the amount of tissue removed and preclude comparison between reports. The ESGS classification divides the parenchyma into five levels and requires that the operation be described by the levels resected together with any non-parotid structures removed [12]. The procedure performed here, a parotidectomy of levels I, II and IV, describes precisely a resection of the entire lateral compartment together with the deep superior compartment that harbored the extension through the stylomandibular tunnel, while sparing the deep inferior compartment. Validation against a large operative series confirmed that this system captures the extent of resection more accurately than conventional nomenclature [25]. Its adoption in reports of vascular anomalies of the parotid gland would allow the extent of resection to be correlated with residual disease and with functional outcome, which is at present impossible.

All branches of the facial nerve were found within the substance of the malformation, and continuous monitoring was used throughout. Meta-analysis of monitoring in parotid surgery indicates a reduction in immediate postoperative weakness with no demonstrable effect on permanent palsy [26]. In a lesion that abolishes normal tissue planes, the value of stimulation lies in identification of branches in an unexpected position rather than in prediction of long-term function. The absence of any deficit in this patient contrasts with several published parotid cases in which excision, or even aspiration, was followed by weakness of the oral commissure and incomplete eye closure [6,13]. Reconstruction of the bed addressed the complaint that brought the patient to operation. Elevation of a superficial musculoaponeurotic system flap over the residual gland restores a barrier between the cut surface of the parotid and the skin flap, reduces the contour deformity and lowers the incidence of gustatory sweating [27]. Excision of redundant preauricular skin, stretched by three years of growth of the lesion, achieved a result comparable with a limited rhytidectomy.

Systemic pharmacological therapy needs to be positioned correctly for this site. Sirolimus produces partial responses in the majority of treated patients with complicated vascular anomalies, and alpelisib has reduced malformation volume and the number of invasive procedures in children with PIK3CA-associated head and neck lymphatic malformations [28,29]. These agents are indicated for extensive, functionally threatening and unresectable disease, and a localized unilateral lesion in an otherwise well adolescent whose principal complaint is facial asymmetry is not an indication for systemic treatment of indefinite duration. Their rational role in parotid disease is neoadjuvant reduction in an extensive lesion before planned surgery, and salvage of progressive residual disease after operation. The present patient remains a candidate for the second strategy should the residual tissue enlarge. Molecular analysis was not performed here. Because genotype increasingly guides the choice of pathway-directed therapy, molecular confirmation of a PIK3CA variant is now encouraged before a PI3K inhibitor is initiated, and tissue-based testing is best obtained at the first resection rather than by a later biopsy [2,29].

The principal postoperative complication was sialocele rather than facial nerve dysfunction. Sialocele and salivary fistula follow any operation that leaves a cut surface of parotid parenchyma beneath a skin flap, with reported incidence between approximately 4 and 27 per cent, and partial resections that leave a larger residual secretory surface carry a higher risk than near-total resections [30]. Most collections resolve with aspiration, pressure dressing and dietary measures, and anticholinergic agents and botulinum toxin type A have become the preferred second-line measures [31]. Reoperation was chosen here because the collection persisted for three months despite conservative management and because imaging and histology had already established that malformation tissue remained within the deep lobe. The second procedure therefore eliminated the secretory source of the pseudocyst and removed a further volume of malformed tissue in one setting. In retrospect an earlier attempt at chemical denervation would have been reasonable, and botulinum toxin should be considered before revision surgery whenever no residual malformation independently requires removal. In this patient, however, botulinum toxin was not administered before revision, because residual malformation within the deep lobe independently required excision, and a single revision addressed both the pseudocyst and the residual tissue. Facial palsy, which dominates the complication profile of surgery for cervicofacial lymphatic malformation, did not occur [32].

Histology reported malformation reaching the radial line of resection focally in both specimens. In oncological surgery this would indicate incomplete resection. In a benign infiltrative vascular anomaly, the interpretation differs, because a clear margin can be obtained only by removing structures beyond the lesion, which at this site means sacrifice of facial nerve branches and of the remaining parenchyma. Acceptance of a focally involved margin is a deliberate consequence of a nerve-preserving strategy and should be reported as such. The corollary is that residual disease must be documented and monitored, since recurrence correlates strongly with completeness of removal. Imaging at 15 months showed narrow bands of high T2 signal at the stylomandibular tunnel and the deep lobe, corresponding in location to the extension identified before operation and to the tissue deliberately not pursued into the parapharyngeal space, without increase in extent or caliber and without clinical manifestation. Radiological residuum and clinical recurrence are not equivalent, and a stable thin band of lymphatic tissue that produces neither deformity nor functional deficit does not justify a second dissection of a previously operated facial nerve.

Selected published reports of lymphatic malformation of the parotid region are summarized in Table 2. Reported patients span the whole age range, surgery was the treatment in almost every case, the extent of resection was described in conventional rather than standardized terms, facial nerve deficit was frequent, follow-up was short and postoperative imaging was rarely reported. The present case differs in the extent of the lesion, in the involvement of both lobes with parapharyngeal extension, in the use of standardized operative nomenclature, in the absence of facial nerve morbidity and in the availability of imaging follow-up. It resembles the published experience in the infiltrative histology, in the difficulty of achieving a clear margin and in the persistence of residual tissue. The limitations are those of a single observation. Follow-up of 19 months is short in relation to a malformation that may expand after infection or bleeding at any point, the residual tissue was assessed qualitatively rather than volumetrically, and genotyping was not performed. Functional and esthetic outcomes were not captured with validated patient-reported outcome measures, quality-of-life instruments, facial symmetry scoring or a validated scar scale, and facial nerve function was graded only with the House-Brackmann scale. The parameters of intraoperative nerve monitoring were not recorded.

## 4. Conclusions

Lymphatic malformation of the parotid and parotideo-masseteric region is a rare infiltrative benign lesion through which the facial nerve runs and in which the glandular parenchyma is involved, so that neither extracapsular dissection nor uniform distribution of a sclerosant is achievable, and uniform sclerosant distribution was considered technically difficult and unlikely in this particular lesion given its infiltrative architecture. Facial nerve-preserving parotidectomy, classified in standardized (ESGS) terms, preserved facial nerve function and restored facial symmetry on clinical assessment. A focally involved margin and a thin, stable residual lesion reflect the deliberate limits of a nerve-preserving strategy and represent residual disease rather than recurrence, and the esthetic result was tempered by a hypertrophic scar. Stable residual tissue at 15 months does not establish the absence of future progression. The risk of local recurrence cannot be established from a single case and requires longer follow-up.

## Figures and Tables

**Table 1:** Clinical timeline of diagnosis, treatment and follow-up.

Time Point	Event
Early childhood	Left parotid/parotideo-masseteric swelling first noted; clinical observation without intervention.
Preceding 3 years	Progressive enlargement with increasing left facial asymmetry; no pain, no bleeding, no infection.
December 2024 (admission)	Magnetic resonance imaging: mixed, predominantly macrocystic malformation 47 × 20 × 55 mm involving the superficial and deep lobe with parapharyngeal extension through the stylomandibular tunnel.
11 December 2024	Primary surgery: Facial nerve-preserving parotidectomy of ESGS levels I, II and IV under continuous nerve monitoring (Inomed C2); SMAS reconstruction. Histology: Cavernous lymphangioma, CD31/D2-40 positive, margin focally involved. House-Brackmann grade I.
Early postoperative period	Salivary retention forming a pseudocyst (sialocele); conservative management.
3 months after primary surgery	Revision: Excision of the pseudocyst with a fragment of the deep lobe; small residual lymphangioma at the radial margin. Facial nerve function normal; no further collection.
15 months after primary surgery	Control MRI: Thin residual bands at the stylomandibular tunnel and deep lobe, no expansion, no recurrence.
19 months (final assessment)	Symmetric facial contour, House-Brackmann grade I in all branches, hypertrophic preauricular scar, no gustatory sweating, no functional complaint.

**Table 2:** Published reports of lymphatic malformation of the parotid region.

Report	Age, Sex	Extent	Treatment	Reported Outcome
Takato et al. 1984 [7]	5, female	Parotid gland	Excision	Infiltration of glandular parenchyma emphasized
Tsui and Huang 1996 [6]	10, male	Left parotid, rapidly enlarging	Aspiration, then observation	Facial palsy after aspiration, resolved with spontaneous regression
Morgan et al. 1997 [5]	35, male	Parotid, cavernous	Excision	Infiltrative growth within the gland
Henke et al. 2001 [14]	34, male	Parotid	Aspiration cytology then excision	Cytology of limited diagnostic value
Mandel 2004 [33]	21, male	Parotid area	Excision	Diagnostic delay emphasized
Naidu and McCalla 2004 [15]	91, female	Head and neck including parotid region	Excision	Review of adult presentation
Imaizumi et al. 2014 [13]	7, male	Parotid	Surgical management	Presented with facial nerve paralysis
Khamassi and Mahfoudhi 2015 [8]	15, male	Lower pole of parotid, 43 × 20 mm, intracystic hemorrhage	Conservative surgery	Extensive and infiltrative behavior emphasized
Chinnakkulam Kandhasamy et al. 2018 [16]	50, female	Superficial lobe, 5 × 5 cm	Excision of cyst	No recurrence at 6 months
Fuad et al. 2023 [9]	8, male	Parotid, haemorrhagic after blunt trauma	Aspiration then surgery	Facial nerve palsy at presentation
Present case	16, male	Superficial and deep lobe, parotideo-masseteric, parapharyngeal extension	Parotidectomy ESGS levels I, II and IV, nerve monitoring, SMAS reconstruction, revision for sialocele	House-Brackmann I throughout, stable residuum on MRI at 15 months, follow-up 19 months

ESGS, European Salivary Gland Society; MRI, magnetic resonance imaging; SMAS, superficial musculoaponeurotic system.

## Data Availability

The original contributions presented in this study are included in the article. Further inquiries can be directed to the corresponding author.
